# Deep-Learning-Based Adaptive Symbol Decision for Visual MIMO System with Variable Channel Modeling

**DOI:** 10.3390/s22197176

**Published:** 2022-09-21

**Authors:** Jai-Eun Kim, Tae-Ho Kwon, Ki-Doo Kim

**Affiliations:** Department of Electronic Engineering, Kookmin University, Seongbuk-gu, Seoul 136-702, Korea

**Keywords:** adaptive symbol decision, channel modeling, deep learning, visual MIMO, generalized color modulation (GCM)

## Abstract

A channel modeling method and deep-learning-based symbol decision method are proposed to improve the performance of a visual MIMO system for communication between a variable-color LED array and camera. Although image processing algorithms using color clustering are available to correct distorted color information in a channel, color-similarity-based approaches are limited by real-world distortions; to overcome such limitations, symbol decision is defined as a multiclass classification problem. Further, to learn a robust classifier against channel distortion, a deep neural network learning technique is applied to adaptively determine symbols from channel distortion. The network designed herein comprises the channel identification and symbol decision modules; the channel identification module extracts a channel identification vector for symbol determination from an input image using a two-dimensional deep convolutional neural network (CNN); the symbol decision module then generates a feature map by combining the channel identification vector and information on adjacent symbols to determine the symbol via learning correlations between adjacent symbols using a one-dimensional CNN. The two modules are connected together and learned simultaneously in an end-to-end manner. We also propose a new channel modeling method that intuitively reflects real-world distortion factors rather than the conventional additive white Gaussian noise channel to efficiently train deep-learning networks. Lastly, in the proposed channel distortion environment, the proposed method shows performance improvement by an average of about 41.8% (up to about 54.8%) compared to the existing Euclidean distance method, and about 6.3% (up to about 9.2%) on average compared to the SVM method.

## 1. Introduction

Visible light communication (VLC) is a wireless technology that transmits information by modulating data through the fast on–off switching of a visible light source. As the information and communication technology (ICT) industry’s demand for high-speed wireless access increases with an increase in data, the competition for occupying low-frequency bands increased, and many studies on VLC were performed to address this problem [1,2,3,4]. In addition, high-power, light-emitting diodes (LEDs) have developed significantly, enabling high-data-rate VLC networks. As a result, VLC has been highlighted as a new paradigm of wireless communication for innovations of the future.

Since most of the currently commercialized VLC-based services are modeled on lighting infrastructure inside buildings, it is clear that there are limits to realizing a hyperconnected internet, which is the ultimate goal of the Internet of Things (IoT) technology [5,6]. For VLC-based IoT technologies to be applied in various fields, such as the automobile, entertainment, and advertising industries, more advanced forms of VLC with improved flexibility and scalability are needed. To overcome the limitations of existing VLC technologies, the generalized color modulation (GCM) technique that secures the flexibility of LED colors and visual multiple-input multiple-output (visual-MIMO) technology that suggests infrastructure scalability through communication between an LED array and camera were developed [7,8,9,10,11,12,13]. In the visual MIMO concept, multiple transmit elements of a light-emitting array (LEA) are used as transmitters to communicate to the individual pixel elements of the camera that act as multiple receive elements to create the visual MIMO channel [11].

Color-independent visual MIMO technology, which combines the GCM method with visual MIMO technology, is expected to be applicable in more diverse fields because it enables continuous communication regardless of changes to the color or brightness of the LEDs during communication [14,15,16]. However, analyzing a signal received through a camera image sensor is not an easy task in the real world; this is because currently available commercial image sensors are very sensitive, and even small changes in external factors such as natural/ambient light, or internal factors such as exposure and focus of the camera, can greatly distort the transmission signals.

In previous studies based on the GCM technique, the Euclidean distance method that calculates the color similarity between the transmitted and received symbols was mainly used to determine the received symbol [10]. The Euclidean distance method was most often used in early studies of the GCM technique, where a single photodiode (PD) was used as the receiver; on the other hand, in the color-independent visual MIMO system, wherein an image sensor is used as the receiver, a color clustering algorithm is applied by taking advantage of the multiple inputs that somewhat guarantee the performance of the Euclidean distance method [17,18]. However, the color clustering algorithm is a classical image processing algorithm that is too simple compared to modern image recognition and computer vision solutions that combine numerous machine-learning and deep-learning algorithms [19]. As the position of the transmitted symbol for calculating the color similarity is fixed, the Euclidean-distance-based symbol decision method cannot respond adaptively when the position of the received symbol deviates greatly from that of the transmitted symbol in situations where the channel distortions change.

Although machine-learning (deep-learning) algorithms in the image processing field have recently developed significantly, classical methods (e.g., color clustering, Euclidean distance) have been mainly used for visual MIMO systems that have to determine color symbols. These methods not only have poor performance, but also do not flexibly respond to channel distortion in various environments. For this reason, in order to improve the SER performance of existing symbol decision methods in visual MIMO, in this study, we propose a method to adaptively determine symbols without additional information, using a deep-learning neural network through the output information of the image sensor, which is the receiver of the visual MIMO system. The whole network to be learned is divided into two modules. The first module is a channel identification module (CIM) that identifies the channel environment from the input image, and the second module is a symbol decision module (SDM) that classifies each symbol using information from its adjacent symbols. These two modules are combined into a single network and learned in an end-to-end manner. In addition, this study presents a new channel modeling method that intuitively reflects the real-world channel distortion characteristics, so that the proposed deep-learning neural network can learn robustly against various real-world channel distortions.

The remainder of this paper is organized as follows. Section 2 presents the proposed deep-learning neural network architecture designed for the adaptive SDM and explains the network training. Section 3 presents the limitations of the existing channel modeling methods and describes the approach that reflects the real-world distortions when performing simulations and experiments. Section 4 compares the symbol error rate (SER) performances of the proposed and existing methods and examines the experimental results. Finally, Section 5 presents the conclusions of this work.

## 2. Deep-Learning Neural Networks for Adaptive Symbol Decision

### 2.1. Deep-Learning Neural Network Architecture

**Figure 1:**
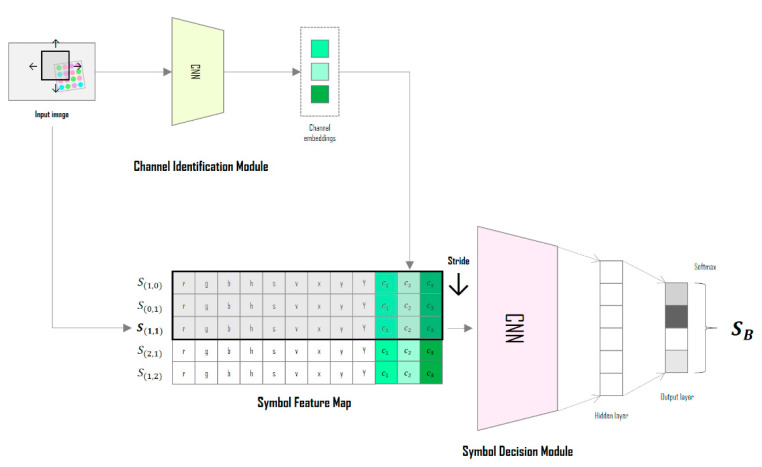
Conceptual diagram of the deep-learning neural network in the proposed method.

Figure 1 shows the overall structure of the deep-learning neural network designed in this study; the network uses the CIM to predict the channel environment from the input image, and the SDM classifies these symbols by learning the correlations between adjacent symbols. Both modules are designed based on the convolutional neural network (CNN) algorithm, which has been mainly used for object recognition, semantic segmentation, and image reconstruction [20,21,22,23,24,25]. These two modules are combined into a single network and are learned in an end-to-end manner.

In the symbol feature map of Figure 1, the bold represents the target symbol (color) to be determined and the different colors mean the different channel identification vectors from CIM.

### 2.2. Channel Identification Module

**Figure 2:**
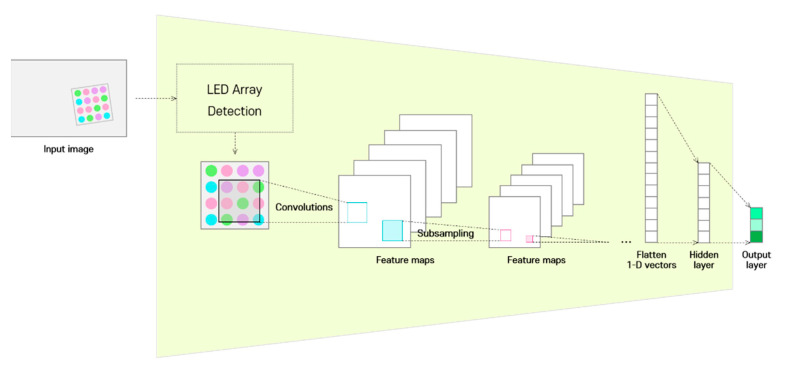
Structure of the channel identification module.

Figure 2 shows the detailed structure of the CIM; the CIM detects the LED array from the image acquired through the image sensor and uses only this image as the input. In a previous study on color-independent visual MIMO systems [26], a technique was proposed for detecting the LED array region in an image, and it was proven that robust and continuous tracking of the LED array is possible through the Kalman filter algorithm.

CIM does not analyze the temporal information of continuous images on the receiver side to identify the channel state, but rather makes use of the spatial information of images obtained through LED array detection at a specific time. The detailed procedure is as follows. The detected LED array region is down-sampled through a two-dimensional CNN, and the compressed information is represented as a vector. In the CIM, the compressed information (feature maps) is transformed to a flattened one-dimensional vector and transferred to the hidden layer through the fully connected layer. This is different from that delivered to the decoder through a fully connected neural network in existing CNN encoder–decoder frameworks. Finally, information is extracted as a short-length implied vector at the output layer, which will henceforth be referred to as the channel identification vector.

The CIM is learned by an implicit method, so that the extracted channel identification vector becomes a feature for minimizing the loss function value of the entire deep-learning model. The final output predicted by the deep-learning neural network in this study is a multi-symbol index, and the approach here is to adaptively solve the multi-symbol classification problem under various channel environments. That is, the CIM is trained to extract additional features that enable classification of multiple symbols robustly based on changes in the channel environment.

### 2.3. Symbol Decision Module

**Figure 3:**
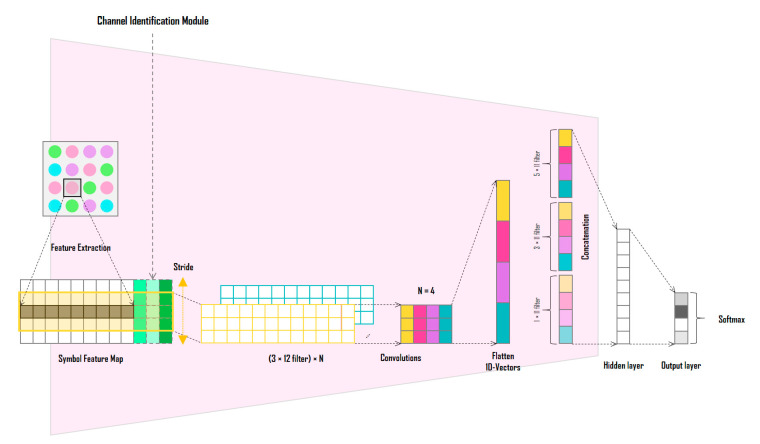
Structure of the symbol decision module.

**Figure 4:**
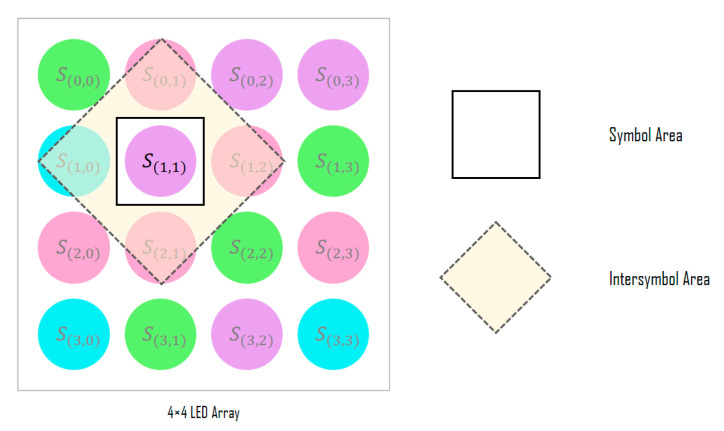
Adjacent symbol area used for symbol decision.

Figure 3 shows the detailed structure of the SDM; the SDM determines the symbol for each LED of the input LED array through signal analysis. First, the channel information of the RGB, HSV, and CIE 1931 xyY models for the corresponding LED position is extracted from the image sensor and combined with the channel identification vector to generate its symbol representation. In the color-independent visual MIMO system, since the LED array is received simultaneously in a single frame from the image sensor, the spatial correlations between adjacent symbols can be important feature information. Therefore, the information of the inter-symbol area is used to decide each symbol, as shown in Figure 4.

The purpose of the Euclidean distance method, which is mainly used as the symbol decision method in existing color-independent visual MIMO systems, is to distinguish the similarity (or difference) between the color of the symbol sent from the transmitter and that of the symbol received at the receiver; thus, the symbol decision method proposed herein determines the symbol with the highest probability by learning its correlations with the adjacent symbols, without calculating the similarities between the two colors in the transmission/reception process.

**Figure 5:**
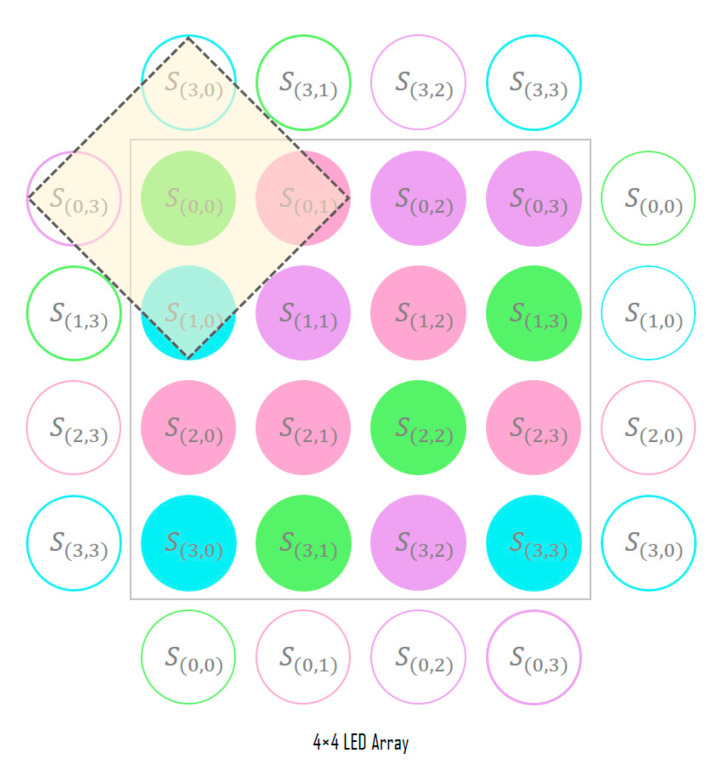
Adjacent symbol area at the LED array boundary.

At the boundary of the LED array, the padding method shown in Figure 5 is used. Although zero padding that fills with zero values or copying the values at the boundary can be used, the degree of distortion received by the LED array from the channel in this study is learned through the symbol correlation information within the LED array. To allow this, the adjacent symbol area is determined by copying the symbol information from the opposite side of the boundary.

**Figure 6:**
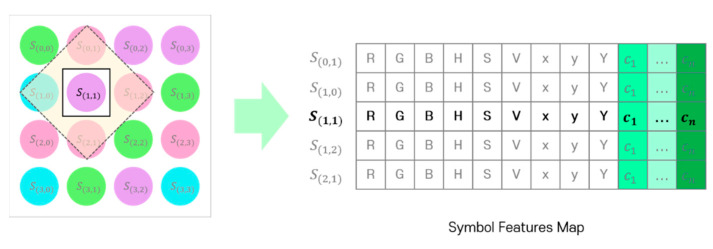
Symbol feature map generated using the adjacent symbol area.

Finally, the feature map required to learn the SDM is formed by combining the representations (RGB, HSV, and CIE 1931 xyY model channel values with channel identification vector) of the symbols included in the adjacent symbol area. Figure 6 shows the symbol feature map generated by the proposed method. The bold part in the figure indicates a symbol to be determined and the corresponding channel information of the RGB, HSV, and CIE 1931 xyY models.

The symbol feature map is used as the input to a one-dimensional CNN based on each feature channel. The network in Figure 3 is a CNN with N filters of size 5 × 12 symbol feature maps and a kernel size of 3. When the symbol feature map passes through one filter, a vector of length 3 is generated; when four filters are used, a vector of length 12 is obtained at the output. Unlike the CNN of the CIM, the CNN of the SDM proposed in this study is not designed as a deep CNN. In [27], the validity of using each channel of the RGB, HSV, and CIE 1931 xyY models as symbol decision features is demonstrated through the SER performance comparison experiment according to the color model. In the experiments, the three color models show almost similar SER performances. In other words, a deep CNN structure is not required because the symbol feature map generated through the adjacent symbol domain can be considered as already feature engineered with domain expertise from the color-independent visual MIMO system and color modeling perspective. Instead, N one-dimensional convolutional filters are applied with different kernel sizes and concatenated to finally extract a one-dimensional vector. The extracted vector passes through a fully connected layer and a softmax function to produce the final symbol. The one-dimensional CNN of the SDM is an independent network that does not share parameters with the CNN of the CIM; however, in the process of learning the entire deep-learning neural network by gradient descent method, it plays an important role in connecting the error backpropagation to the CNN of the CIM. In other words, the CNNs of the two modules are organically coupled, so that the error of the SDM is transmitted to the CIM by error backpropagation, and the output of the CIM affects the final output performance of the SDM. Through this process, the SDM learns to determine symbols in various channel distortion environments.

## 3. Variable Channel Modeling

### 3.1. Limitations of Existing Channel Modeling Methods

**Figure 7:**
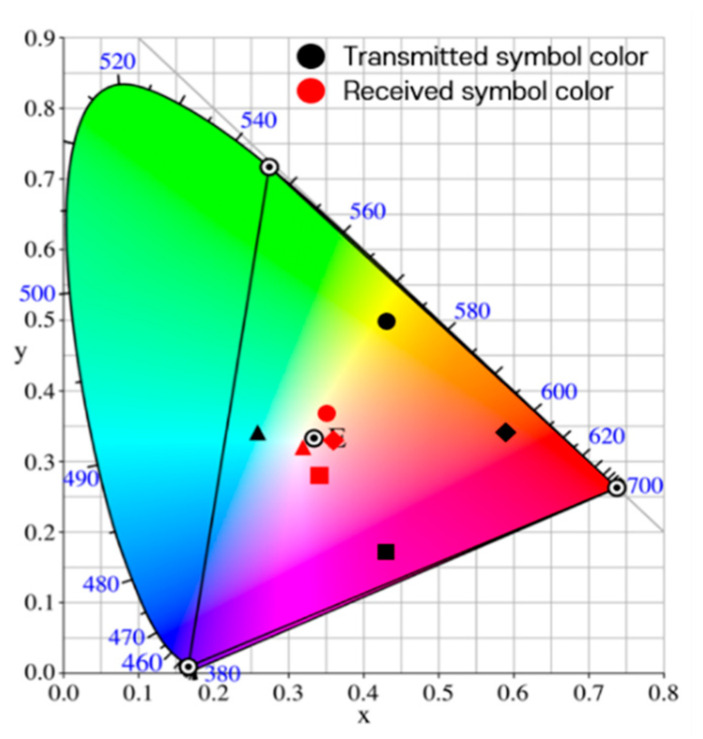
Distortion of the symbol transmission/reception process as observed in the real world.

In previous studies, simulations were performed by adding the values of random variables of a Gaussian distribution defined according to the variance size as noise to each channel of the RGB model [10]. Figure 7 shows the actually observed representative distortions of the real world on the CIE 1931 xy chromaticity coordinate system [17]. In the figure, the symbol ‘⊙’inside the triangle indicates the white color position in the color space, and the symbols ‘⊙’ at the vertices of the triangle indicate the positions of red, green, and blue colors. It is seen that all four types of received symbols are clustered around white. This is a case in which the image captured by the image sensor suddenly becomes bright or dark, and occurs when the saturation of the LED array part of the image is lowered. When the saturation is low, as the brightness increases, the color approaches white, and as the brightness decreases, the color approaches black.

In general, it is difficult to intuitively understand the high and low levels of brightness and saturation in images represented by RGB models. Hence, the HSV model color space, designed to easily analyze the brightness and saturation levels in images, is widely used [28]. Since this study proposes an adaptive symbol decision method based on channel state changes, a channel modeling technique that is more reasonable to the real world is needed to effectively reflect the real-world distortions in deep learning.

### 3.2. Proposed Channel Modeling Method

**Figure 8:**
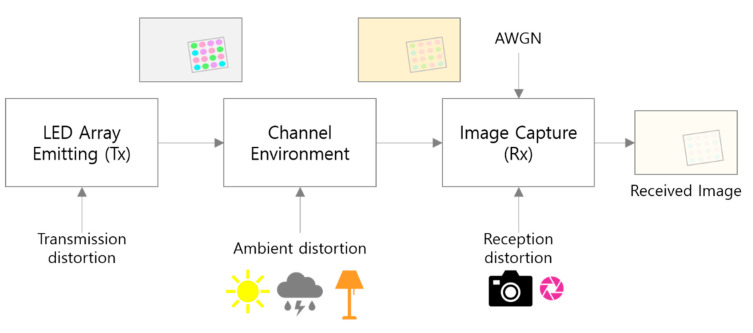
Real-world channel distortion factors in visual MIMO systems.

Figure 8 shows the real-world distortion factors that may occur during the transmission/reception process of the visual MIMO system by classifying them into stages. By classifying these factors of distortion that occur simultaneously in the communication process as shown in Figure 8, it is assumed that the distortion at each step can be limited as follows. Transmission distortion is a factor where the transmitter LED emits light of the modulated symbol color, and it is assumed that it mainly causes color distortion of the symbol, or a distortion phenomenon in which the signal strength of the symbol is attenuated. Next, it is assumed that ambient distortion is a factor that occurs when natural or ambient light is strongly irradiated; this mainly causes color changes to an image and color distortion of the symbols. Reception distortion is a factor that occurs when the camera settings, such as exposure and gain, are adjusted. Finally, additive white Gaussian noise (AWGN) is considered as a channel noise factor of the image sensor output in the RGB model.

We cannot say that these limited assumed distortions reflect all the real-world distortions. In reality, all of the distortions assumed above may occur at the same time during the transmission/reception process. However, since the distortion phenomena assumed above are the biggest challenges for successful commercialization of visual MIMO systems, it is absolutely necessary to reflect them in the channel modeling.

In this study, the channel state according to time that reflects the major distortion factors is modeled as Equations (1)–(3):

(1) $$S_{rx}=C_{t}\left(S_{tx}\right)$$

where $C_{t}\left(\cdot \right)$ denotes a channel state function at time *t* and $S_{tx}$ and $S_{rx}$ denote the transmit and receive symbol vectors in the color space, respectively. The symbol vector *S* can be represented as Equation (2), according to the color space in which it is expressed:

(2) $$S^{\left(space\right)}=\left\{\begin{array}{c} {\left[R,G,B\right]}^{T}\\ {\left[H,S,V\right]}^{T}\\ {\left[x,y,Y\right]}^{T} \end{array}\right.$$

where *R*, *G*, and *B* represent the three channel values of the RGB color space; *H*, *S*, and *V* represent the three channel values of the HSV color space; *x*, *y*, and *Y* represent the three channel values of the CIE 1931 xyY color space. The three color spaces in Equation (2) are modeled to be mutually transformable based on the RGB model [28,29]. The formulas for conversion between these color spaces is detailed elsewhere [28,29].

The channel state function $C_{t}\left(\cdot \right)$ can be recursively represented as in Equation (3), so that the distortion factor is linked according to the transmission/reception flow shown in Figure 8:

(3) $$C_{t}\left(S_{tx}\right)=D_{Rx}\left(D_{Light}\left(A\times D_{Tx}\left(S_{tx}\right)\right)\right)+n_{awgn}$$

where $D_{Tx}\left(\cdot \right)$ is a transmittance distortion function, $D_{Light}\left(\cdot \right)$ is an ambient distortion function, $D_{Rx}\left(\cdot \right)$ is a reception distortion function, *A* is a weight by transmitter attenuation distortion, and $n_{awgn}$ denotes the conventional AWGN for the RGB model.

As described above, the transmission and ambient distortions cause changes in the chromaticity of the symbol color, and the reception distortion can cause changes in the saturation and brightness of the symbol color. Accordingly, Equation (3) can be represented as Equation (4):

(4) $$C_{t}\left(S_{tx}\right)=D_{HSV}\left(D_{CIE}\left(S_{tx}\right)\right)+n_{awgn}$$

where $n_{awgn}$ can be represented as in Equations (5) and (6).

(5) $$n_{awgn}={\left[n_{r},n_{g},n_{b}\right]}^{T}$$

(6) $$n_{r}\sim N\left(0,\sigma_{r}^{2}\right),\;n_{g}\sim N\left(0,\sigma_{g}^{2}\right),\;n_{r}\sim N\left(0,\sigma_{r}^{2}\right)$$

In Equation (4), $D_{CIE}\left(\cdot \right)$ is a function that distorts the chromaticity of the input signal, and is given by Equations (7)–(9).

(7) $$D_{CIE}\left(S_{tx}^{\left(CIE\right)}\right)=S_{tx}^{\left(CIE\right)}+n_{xy}^{\left(CIE\right)}$$

(8) $$n_{xy}^{\left(CIE\right)}={\left[n_{x},n_{y},0\right]}^{T}$$

(9) $$n_{x}\sim N\left(0,\sigma_{x}^{2}\right),\;n_{y}\sim N\left(0,\sigma_{y}^{2}\right)$$

$D_{HSV}\left(\cdot \right)$ is a function that distorts the brightness and saturation of the input signal, and is expressed by Equations (10)–(12).

(10) $$D_{HSV}\left(S_{tx}^{\left(HSV\right)}\right)=S_{tx}^{\left(HSV\right)}+n_{xy}^{HSV}$$

(11) $$n_{sv}^{\left(HSV\right)}={\left[n_{s},n_{v},0\right]}^{T}$$

(12) $$n_{s}\sim N\left(0,\sigma_{s}^{2}\right),\;n_{v}\sim N\left(0,\sigma_{v}^{2}\right)$$

Therefore, the proposed channel modeling technique includes a total of seven variables ($n_{x},n_{y},n_{s},n_{v},n_{r},n_{g},n_{b}$), and each can be variably controlled by a Gaussian distribution with a different variance ($\sigma^{2}$) value. The transmission/reception process shown in Figure 8 is only one scenario for a new channel modeling, and Equation (13) is also just one method defined by this scenario. The key here is that more intuitive and detailed distortion variables should be reflected in the channel model through multiple color spaces, to account for various channel distortions. Therefore, the channel state function through the proposed channel modeling can be represented as a comprehensive expression, in which the total distortion is determined by the seven variables defined above.

(13) $$C_{t}\left(S_{tx}\right)=S_{tx}+f(n_{r},n_{g},n_{b},n_{s},n_{v},n_{x},n_{y})$$

### 3.3. Channel Modeling Verification

**Figure 9:**
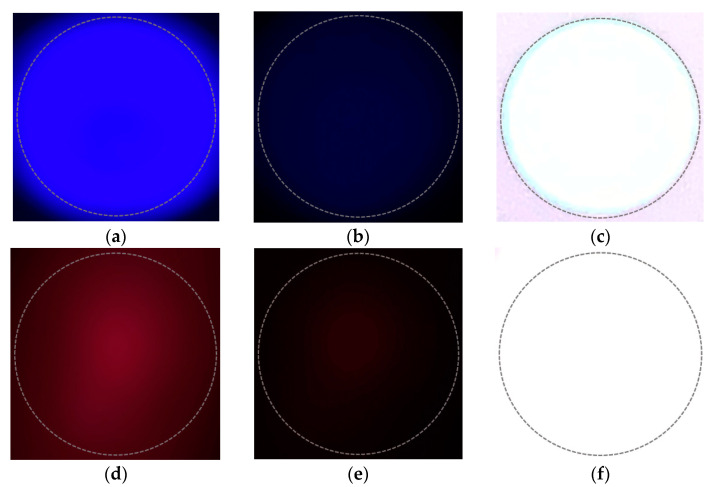
Differences in symbol images according to camera parameter adjustment. (**a**) When exposure is appropriate; (**b**) when exposure is low; (**c**) when exposure is high; (**d**) when exposure is appropriate; (**e**) when exposure is low; (**f**) when exposure is high.

To validate the proposed channel modeling method, we attempt to reproduce the real-world distortions by adjusting the seven variables in Equation (13). Figure 9 shows the differences between the symbol images obtained by adjusting the exposure of the camera in the real world. In the figure, the upper and lower rows are examples of blue and red symbols, respectively. The dotted circle in the figure, represent the shape of a light source (LED). With respect to a camera, exposure refers to the amount of light that enters the image sensor through the lens; a high exposure means that a large amount of light enters the image sensor and a low exposure indicates that a small amount of light enters the image sensor through the lens. Exposure is determined by the camera’s aperture (F) and shutter speed. The smaller the aperture value, the larger the opening of the aperture and amount of light received. The lower the shutter speed, the longer the shutter is open; the longer the image sensor is exposed to light, the greater the amount of light received.

Table 1 shows the observed information in the images of Figure 9 through the RGB, HSV, and CIE 1931 xyY models.

**Figure 10:**
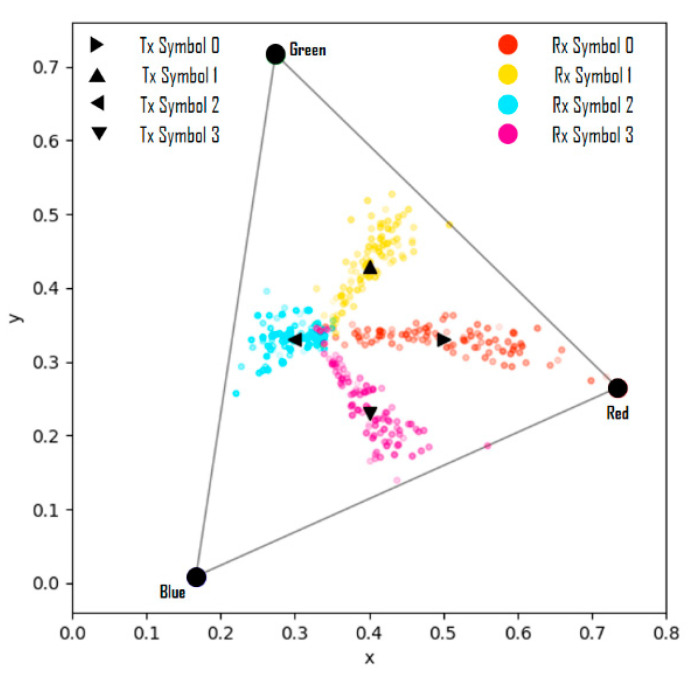
Symbol transmission/reception simulation results from channel modeling.

When the camera exposure is changed, the channel-changing pattern of the RGB model differs depending on the symbol color. However, in the case of the HSV and CIE 1931 models, it is seen that the channel changes have similar trends, regardless of the symbol colors. Thus, it can be considered that the HSV and CIE 1931 models are suitable for channel modeling. A simulation experiment of the transceiving symbols of a 4 × 4 LED array was performed to reproduce the real-world distortions using the proposed channel modeling method. If the seven variables of Equation (13) are set as shown in Table 2, and the coordinates of the symbols received for 100 frames (total of 1600 symbols) are plotted in the CIE 1931 xy chromaticity coordinate system, the results are as shown in Figure 10.

**Figure 11:**
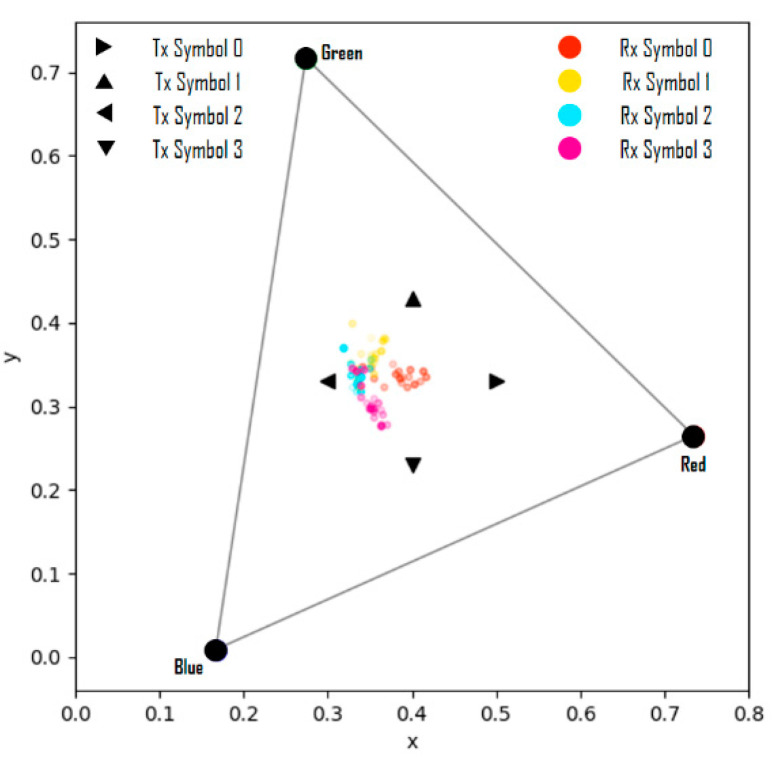
Symbol transmission/reception simulation results from channel modeling. ($n_{s}\leq -0.2$).

In Figure 10, when the values of the variable $n_{s}$ are less than −0.2, the results are shown in Figure 11; these results represent the case where the symbol saturation is lowered and it can be considered as distortion wherein the camera exposure is largely adjusted. Therefore, it is proven that the proposed channel modeling method correctly reflects the real-world distortion factors and reproduces specific distortion phenomena similarly through the parameter settings.

## 4. Simulation and Experimental Results

### 4.1. Experimental Environment

Table 3 shows the GCM information used in the simulations. In the simulations, the LED array of the transmitter was set to a size of 4 × 4, and the 16 symbols were arranged and transmitted in parallel. The constellation diagram comprised four types of symbols through 2-bit encoding modulation. Table 4 shows the color space information for the target colors and symbols used in the simulations.

For the training of the proposed deep-learning neural network, as shown in Table 5, a total of 21 random channel environments (ID 0–20) were defined using the channel modeled in Section 3.2, and a total of 4200 frames (67,200 symbol samples) were generated as training data via 200 frames in each environment. In addition, to increase the reliability of the experimental results, the same amount of verification data as the training data were generated in an environment that was slightly worse than the environment used to generate each step of the training data. Table 5 shows the training data used in the simulation experiment, and Table 6 shows the verification data.

### 4.2. SER Performance

The comparison target for the performance evaluation in this study is the Euclidean distance method used in existing color-independent visual MIMO systems [27]. Additionally, the performance is compared with that of the support vector machine (SVM) method [30]. The SVM, which is already proven in numerous applications requiring machine learning, is the supervised learning model with associated learning algorithm that analyzes data for classification and regression analysis. In the case of SVM as a comparison target, radial basis function (RBF) kernel was adopted, and the values of gamma and C parameters with the highest performance were used experimentally. Here, C is a hypermeter that is set before the training model and used to control error, and gamma is also a hypermeter that is set before the training model and used to give curvature weight of the decision boundary. As a performance comparison metric, the SER is used, and SER performance is calculated as the reciprocal of accuracy.

**Figure 12:**
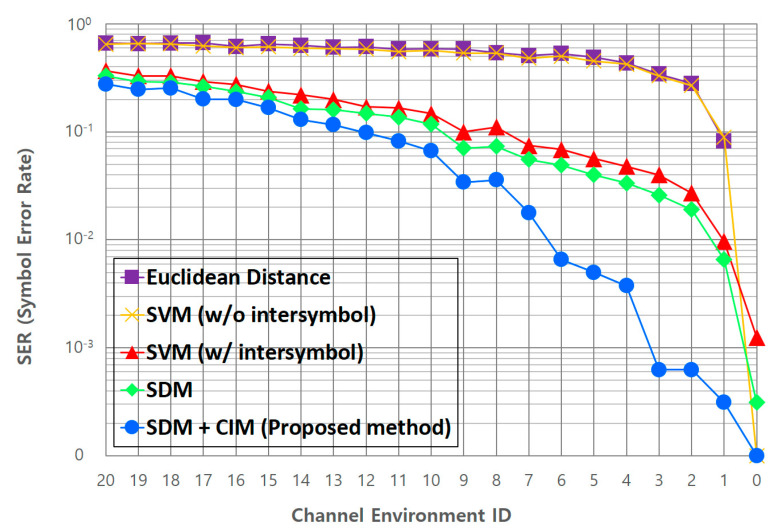
SER performance of the proposed adaptive symbol decision method.

The performances of the Euclidean distance method, which is the existing symbol decision method, and the SVM method, which is a representative machine-learning algorithm, are compared. Then, we compare the SER performance of the proposed deep-learning-based adaptive symbol decision method with that of the SVM method, which shows the best performance before deep learning. Figure 12 shows the SER performance comparisons between the Euclidean distance and SVM, SDM, and CIM + SDM (proposed) methods. The vertical axis indicates the SER value, and the symbol decision performance increases as the SER decreases. The horizontal axis indicates the channel environment ID value specified in Table 6, and the larger the value, the more severe the transmission symbol distortion due to the channel environment. Inside the figure, “w/intersymbol” means that learning with the information of adjacent symbols is considered, while “w/o intersymbol” means that learning with the information of adjacent symbols is not considered.

As seen in Figure 12, in the Euclidean distance method, the symbol decision performance deteriorates rapidly if the channel condition deteriorates even a little. Through this, it is clearly seen that the Euclidean distance method has obvious limitations in the proposed new channel modeling environment. In the case of the SVM method, on average, the performance improves by about 33.4% (up to about 43.6%) compared to the Euclidean method when learning with the information of adjacent symbols proposed in this study. Through this, it is proven that learning the information of adjacent symbols together has a positive effect on the symbol decision performance in situations where the channel is greatly distorted. Next, we compare the SER performance of the SVM and proposed CNN-based SDM methods. Both methods learn the correlations between symbols by using them as information features of the adjacent symbols. It can be seen that the CNN-based SDM shows a better overall performance than the SVM method. Although there is no dramatic performance improvement, the performance is improved by about 2.6% (up to about 5.7%). Considering the total amount of verification data, about 3830 symbol errors are reduced compared to the SVM method. From this, we can conclude that the deep-learning-based method is more suitable for learning various channel distortion factors than the classical machine-learning method. Figure 12 also presents the SER performance of the deep-learning-based adaptive symbol decision method (SDM + CIM) proposed in this study.

When the CIM is combined with the SDM, the performance improves on average by about 3.7% (up to about 6.2%). Among the comparison groups shown in Figure 12, only the proposed method maintains the SER below 1% until the channel environment ID #6. Through this, it is proven that the proposed method adapts to the channel changes and correctly determines the symbol, even in an environment where the channel condition deteriorates rapidly. Overall, it can be seen that the performance of the proposed deep-learning-based adaptive symbol decision method is improved on average by about 41.8% (up to about 54.8%) compared to the existing Euclidean distance method, and by about 6.3% (up to 9.2%) compared to the SVM method in the entire channel state used for evaluation.

## 5. Conclusions

This study proposed an adaptive symbol decision method for a visual MIMO system to which a deep-learning neural network was applied. The proposed deep-learning neural network comprises the SDM and CIM, both of which use the CNN algorithm. The CIM is designed with a deep CNN structure, and the SDM is designed with a one-dimensional convolutional multiple-filter structure. In addition, unlike the existing symbol decision methods that calculate the color similarities between the transmitted and received symbols to distinguish the received symbols, the proposed SDM uses the information of the adjacent symbols as the feature map, and determines the symbols by learning the correlations with the adjacent symbols.

To prove the superiority of the proposed adaptive symbol decision method over extant methods, a new channel modeling technique of a multicolor model mixing method that reflects various real-world distortion factors is presented; based on this, we succeed in reproducing the distortions caused by natural and ambient light and camera exposure variations in the real world. In addition, by directly comparing the performances of the learning methods in the proposed channel modeling environment and in the existing AWGN channel modeling environment, the proposed method is shown to not only include the AWGN environment, but also reflect more realistic channel distortion factors than the AWGN.

Lastly, in the simulations and experiments performed in the proposed channel distortion environment, the proposed method shows performance improvement by an average of about 41.8% (up to about 54.8%) compared to the existing Euclidean distance method, and about 6.3% (up to about 9.2%) on average compared to the SVM method. Based on the presented SER performances, it is proven that the deep-learning-based adaptive symbol decision method proposed in this study can robustly maintain SER performance, even when the channel environment deteriorates rapidly.

## Figures and Tables

**Table 1:** Color space information based on camera exposure changes.

Figure 9	HSV Model			RGB Model			CIE 1931 Model		
	H (°)	S (%)	V (%)	R	G	B	x	y	Y
Figure 9a	246	100	100	26	0	255	0.1691	0.0099	0.0120
Figure 9b	240	100	23.5	0	0	60	0.1670	0.0090	0.0004
Figure 9c	180	9.4	100	231	255	255	0.3151	0.3364	0.9656
Figure 9d	344	87.1	39.6	101	13	37	0.6353	0.2302	0.0243
Figure 9e	352	90.6	12.5	32	3	7	0.6972	0.2537	0.0019
Figure 9f	0	0	100	255	255	255	0.3333	0.3333	1.0000

**Table 2:** Channel modeling parameter settings.

$N\left(0,\;\sigma^{2}\right)$	$n_{x}$	$n_{y}$	$n_{s}$	$n_{v}$	$n_{r}$	$n_{g}$	$n_{b}$
σ	0.01	0.01	0.2	0.2	0.01	0.01	0.01

**Table 3:** GCM information for simulations.

Parameter	Value
Color space	CIE 1931
RGB model	CIE RGB
Reference white	E
LED array size	16 (4 × 4)
Number of constellation points	4
Intensity (Y value)	0.165
Three positions of RGB LEDs in CIE 1931 space	R: (0.735, 0.265) G: (0.274, 0.717) B: (0.167, 0.009)

**Table 4:** Target color and symbol information.

Target Color		Symbol	x	y	R	G	B
x	y						
0.40	0.33	s1	0.50	0.33	168	96	82
		s2	0.40	0.43	118	112	72
		s3	0.30	0.33	97	115	117
		s4	0.40	0.23	168	95	138

**Table 5:** Training data information.

Channel Distortion	Channel Environment ID				
	0	1	…	19	20
Std. of x, y noise distribution	0.0	0.02	**…**	0.38	0.40
Std. of S, V noise distribution	0.0	0.02	**…**	0.38	0.40
Std. of AWGN	0.0	0.02	**…**	0.38	0.40
Number of frames	200	200	**…**	200	200
Sample frames	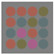 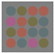	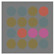 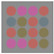	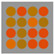 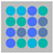	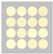 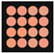	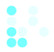 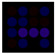

**Table 6:** Verification data information.

Channel Distortion	Channel Environment ID				
	0	1	…	19	20
Std. of x, y noise distribution	0.01	0.03	**…**	0.39	0.41
Std. of S, V noise distribution	0.01	0.03	**…**	0.39	0.41
Std. of AWGN	0.01	0.03	**…**	0.39	0.41
Number of frames	200	200	**…**	200	200
Sample frames	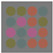 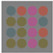	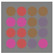 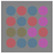	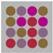 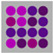	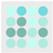 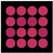	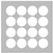 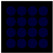

## Data Availability

The dataset used in this research is available upon a valid request to any of the authors of this research paper.
